# Decision-Making, Violence, Resistance, and Love: Contested and Complicating Narratives of Syrian Marriages

**DOI:** 10.1177/10778012231207037

**Published:** 2023-10-12

**Authors:** Michelle Lokot

**Affiliations:** 4906 London School of Hygiene and Tropical Medicine, London, UK

**Keywords:** marriage, refugees, Syria, gender-based violence

## Abstract

Within scholarly literature as well as reports from humanitarian actors, including international nongovernmental organizations (NGOs), United Nations agencies and local NGOs, Syrian marriages are often described in static, essentialist ways that reinforce Orientalist assumptions. Based on feminist ethnographic research with Syrian women and men in Jordan, this article explores marriages in historical and intersectional context, before and during displacement. The article challenges common representations of Syrian marriages and advances how Syrian women's power and agency are understood. It emphasizes women's role in deciding to marry (or not) and discusses violence and love in marriage and resistance to proposed love marriages.

## Introduction

Within research on Middle Eastern families, the relationship between a husband and wife has received significant focus, reflecting the emphasis on the family as a “sacred space” in the region (Joseph, 2000, p. 19). Marriage continues to be recognized as critical to societal functioning and social reproduction in the region while also determining identity and political affiliations (Hughes, 2015, p. 3; Johnson et al., 2009, p. 14). Scholars observe the “nuptiality transition” occurring in the region resulting from social, economic, and educational shifts, pointing out changes in marriage practices over time (Hussein & Manthorpe, 2007, p. 4). Delays in the age of marriage as well as the increased cost of marriage may result in what Diana Singerman (2006, p. 6) calls “waithood,” a liminal state prior to marriage that extends financial dependency on parents, or what An Van Raemdonck (2023) calls “double waithood”—waiting for financial means to marry and for legal residency and resettlement rights.

As a result of the conflict in Syria, the topic of marriage has received greater attention over the last decade, with not only academic but also humanitarian actors such as international non-governmental organizations (NGOs), United Nations agencies, and local NGOs analyzing the meaning and nature of Syrian marriages. This article, in critiquing the Orientalist assumptions that sometimes accompany such analyses, takes an intersectional and historical approach to understanding Syrian marriages, drawing on ethnographic data to complicate common representations of Syrian marriages.

Academic research on Syrian marriages, although limited, reveals complexities in the politics and meaning of marriage. In Syria, marriage is tied to women's position in society. Aligning with narratives from other settings about women's relationship to the state, Syrian women symbolize the nation, with national narratives shifting between women representing tradition or progress (Rabo, 1996, p. 159). Politics and gender are intertwined in popular Syrian soap operas that present the male patriarch as an oppressive dictator, linking marriage to a critique of the Syrian state (Joubin, 2013). Maria Kastrinou (2016, p. 1), in her research among the Druze in Damascus, describes how politics infuses marriage: “Marriages, in most cultural contexts, are pivotal transformative rituals that sanction and appropriate unions, that perform and embody the social reproduction of communities and society at large.” Christa Salamandra (2006, p. 161) who conducted research among upper-class Damascenes, also comments on the importance of marriage: “Marriage, with the alignment of families, the melding of cultural and economic capital it entails, is a primary locus of identity and sociability.” Marriage may involve “a union of families” (Bennett, 1999, p. 123) or be a means of establishing social relations with “outsiders” (Rugh, 1997, p. 241). Bouthaina Shaaban's (1991) personal account as a Syrian woman illustrates how family resistance may result in having to choose between family and marriage. She suggests that the religious and nationality differences between her and her proposed spouse cited by her father and brother were “only the pretext” to their resistance compared to the fact that she chose her partner without their guidance (p. 6).

In academic research on Syria, there is recognition of the tensions between stereotypical depictions of women's roles alongside examples of women exercising agency and decision-making power (Chatty, 1986, 2018; Gallagher, 2012). Salamandra (2006) also suggests that marriage is the point at which “old ideals and new realities” are held in tension, pointing to the potential for challenges to gender norms. Annika Rabo's (2008) ethnographic research in Raqqa reveals complicating narratives of women's power within marriages, including accounts of women's control of economic resources. Despite this different narrative about women's power, women continue to receive blame for men's sexual transgressions (pp. 151–152). Similarly, Andrea Rugh's (1997, p. 74, 235) ethnographic research in a village outside Damascus demonstrates both practices that appear “atypical” within depictions of marriage in Arab contexts, such as women making decisions about major household purchases, as well as on the other hand, the maintenance of distinct gender roles. In some respects, marriage might be viewed as a potential site for social change, where relationships evolve and where gender norms may be resisted (Salamandra, 2006) while in other cases power and norms remain unchanged.

Within historical research on marriage in the Middle East context more broadly, marriage is sometimes represented in static ways. “Traditional” marriages are framed as involving passive actors and lack of love, in contrast to modern marriages which are shaped by Westernization and individualization and therefore involve romance (Hart, 2007, p. 346). Scholars have critiqued depictions of Arab brides as passive and subservient as well as Western feminist assumptions that such characteristics are automatically linked to Islam (Bennett, 1999, p. 161). Lila Abu-Lughod (2013, p. 95) critiques negative depictions of Muslim culture, suggesting that the public fascination for “sordid” narratives about women is “disquieting.” She argues narratives must not simplistically blame culture. This is echoed by others who argue that the notion of a passive Arab bride “overshadows real-life struggles” that Syrian women face in aligning ties and loyalties after marriage (Bennett, 1999, p. 161). Mansour et al. (2014, p. 537) critique assumptions made by policy-makers about marriage and sexual practices of Bedouin communities in Lebanon, suggesting these are based on “stereotypical and Orientalist” perspectives.

Within the literature on marriage in this region, there is a noted absence of research focused on the topic of love (Adely, 2016, p. 105). Abdallah's (2009, pp. 58–59) research on marriage among Palestinian refugees suggests love is sometimes viewed as both “utopian” as well as a “social threat.” Fuad Khuri (2007, pp. 23–25) observes that love is more acceptable for Arab women than Arab men because men will not admit to being in love. These depictions are not neutral; representations of sexual repression and the absence of emotion or love reinforce colonial messaging about the need for more modern relationships (Fortier et al., 2016, pp. 96–97). Scholars have urged nuance in depicting marriage, suggesting that love and male domination may coexist (Muhanna, 2013, p. 18). Research among Palestinian refugees in Jordan reveals marriages are based on the consolidation of family ties or “pragmatic” reasons, leaving love to the periphery (Abdallah, 2009, p. 50). Fida Adely's (2016) research in Jordan suggests compatibility may be more important than romantic love. Cousin marriage, rather than marriage for love has often been a focus of research in the region (Rabo, 2008, p. 129). Rosemary Sayigh's (1993, pp. 267–269) landmark research in Shatila camp in Beirut found women “editing out” their marriages in sharing life stories and excluding husbands as topics for discussion. Suad Joseph's (1994a, pp. 57–60) research in Camp Trad in Beirut emphasized the role of sibling relationships over marital relationships, suggesting familial ties may supersede marital ones. In research in this region, men are positioned as powerful, with uncontrollable sexual urges (Hasso, 2011, p. 114). Nasser El-Dine's (2018) research in Amman emphasizes that marriage involves entanglements of both love and money; meeting the material requirements for marriage does not negate love. Kastrinou (2016) discusses the presence of both intimacy and violence within marriages among the Druze of Syria, outlining how class and sectarian politics infuse marital relations. Kimberly Hart (2007, p. 354), based on research in Turkey, suggests that the division between love marriage and arranged marriages is “increasingly blurry.” Others argue marriage is not always the sole focus of women or men, for example, Johnson (2010, pp. 109–110) found that for Palestinian women, education was a marker of modernity; women preferred to marry in their early to mid-20s instead of earlier as commonly assumed. Marriage may also be a “collective aspiration” for refugees (Zbeidy, 2020, pp. 142–146).

These descriptions of marriage align with conceptualizations of gender norms as locational, cultural, and changing over time. “Gender norms,” which are the expected, ideal behavior of women and men, are not fixed, rather power is always shifting, varying based on age, class, and other power structures (Connell, 2005, p. 76). Gender norms correspond to the way gender—a system of power describing how “social practice” may be “ordered” (Connell, 2005, p. 71)—is constructed within a particular context. The gender order is shaped by patriarchy. Speaking of the Arab context, Joseph (1999, p. 12) writes: “Patriarchy entails cultural constructs and structural relations that privilege the initiative of males and elders in directing the lives of others.” Joseph's conceptualization of patriarchy is often situated within family relationships (1993), however, scholars like Marcia Inhorn (1996, p. 4) observe that patriarchy is not uniform across Arab, Middle Eastern, or Muslim settings, nor is it only visible within family structures: “women's subordination is first experienced – sometimes subtly, sometimes profoundly – within the family, which serves as a template for the reproduction of patriarchal relations in other realms of social life.” Debates about the meaning and nature of patriarchy include consideration of how patriarchy intersects with neoliberalism and colonialism, as well as how patriarchy may not be the sole cause of women's oppression (Abu-Lughod, 2013; Kandiyoti, 1988; Patil, 2013). Patriarchy is not always easy to understand (Abu-Lughod, 2013, p. 3). Analyses of patriarchy within the Middle East context have included recognition that patriarchy and submission should not be viewed as a binary (Mahmood, 2001, p. 205).

Scholars have examined how gender norms and patriarchy impact displacement (Grabska, 2014, p. 200), emphasizing that displacement may challenge or heighten gender inequalities, or even result in changes in both directions (El-Bushra, 2003; Matlou, 1999). Grabska (2014, p. 200) writes: “War and displacement give rise to multiple forms, both weakened and reinforced, of femininities and masculinities.” Refugees may exercise new authority due to displacement, for example, young men may have more power than older men during displacement and may move into leadership roles (Grabska, 2014, p. 86; Turner, 1999, p. 145). Humanitarian narratives have chosen to fixate on the relationship between gender and displacement, using issues like early marriage and gender-based violence to demonstrate how displacement disrupts the normal gender order (Oxfam & ABAAD, 2013; UNHCR, 2014). In the case of Syria, scholars have demonstrated that early marriage was a longstanding feature, rather than solely a result of displacement. Margaret Meriwether's (1999) analysis of family dynamics in Ottoman Aleppo suggests early marriage among Syrians within that period should be viewed in context—as linked to differing constructions of childhood and adulthood which prescribed that girls and boys took on adult responsibilities during puberty. Her argument points to the fact that historically, early marriage in Syria may reflect different understandings of adulthood among Syrians—rather than Western ideas about marriage.

International NGOs, United Nations agencies, and local NGOs have also been engaged in analyses of Syrian marriages, specifically since the conflict began in 2011. Within reports focused on gender and Syrian refugees, narratives have often centered on how displacement has resulted in more “early marriages” (International Rescue Committee, 2012; Oxfam & ABAAD, 2013; Save the Children, 2014). Early marriage is commonly understood as marriage occurring under the age of 18 when it is understood that consent is not possible (Al Akash & Chalmiers, 2021). Early marriage is not depicted as influenced by love but shaped by economic pragmatism, or the need to protect girls from violence. Such marriages are often described as forced upon girls, especially by fathers (Al Akash & Chalmiers, 2021; Higher Population Council—Jordan, 2017; Save the Children, 2014; UNICEF, 2014)—a narrative which links to humanitarian representations of refugee men as “primitive” and requiring reform (Olivius, 2016, p. 56). Scholars assert that linking early marriage to patriarchy can negate the role of other factors in sustaining the practice and perpetuate racialized assumptions (Zuntz et al., 2021, p. 183).

Apart from the focus on early marriage within humanitarian reports, Syrian marriages more generally are often presented as characterized by tension associated with displacement, including housing expenses, barriers to accessing work, and protection risks. Syrian women are said to experience violence, which is attributed to societal disruptions caused by displacement (International Rescue Committee, 2014, p. 5; UN Women, 2013, p. 10; World Vision International, 2020). Some academic scholars have critiqued these representations, drawing attention to the problems associated with positioning women and girls as perpetually vulnerable and requiring empowerment or rescue from humanitarian aid (Hyndman, 2004; Van Raemdonck & De Regt, 2020; Zbeidy, 2020; Zuntz et al., 2021). Humanitarian analyses have been critiqued for reinforcing common generalizations while positioning refugees as “other” (Al-Ali, 2016, p. 3; Turner, 2017, p. 45). Lila Abu-Lughod's recent (2021, p. 289) critique of the gender-based violence agenda echoes her (1989) criticism of those who “unwittingly partake in a colonial discourse” about women in the Middle East—a critique which may apply to both academics and humanitarian actors.

In exploring marriages among Syrians in a historical and intersectional context, this article deviates from dominant analyses of Syrian marriages which focus solely on displacement, by presenting the negotiations, conflicts, and power dynamics surrounding marriage before and during displacement. This article is based on findings from feminist ethnographic research. It unravels two common conceptualizations of Syrian marriage. Firstly, it challenges depictions of Syrian marriages as solely shaped by the decisions of older men in a family, drawing attention to multiple influences upon marriage decisions. Secondly, it diverges from dominant representations of Syrian marriage as primarily characterized by violence, drawing attention to experiences of violence within marriage, love within marriages, and resistance to proposed love marriages.

## Methodology

Research was conducted in Jordan over 9 months in 2016–2017 to explore family relationships, mobility, and gender roles. Self-settled Syrian refugees living in Zarqa, Irbid, Jerash, and Amman participated in the research. They were largely from Dara’a, Damascus, and Homs in Syria and tended to be middle-class, with a few exceptions, which are discussed in the article. The research methods included participatory photography, semistructured interviews, life story interviews and participant observation with Syrian women and men aged 18–60, and semi-structured interviews with humanitarian workers. In this article, analysis from life story interviews, semistructured interviews and focus group discussions (FGDs) with Syrian women and men are discussed.

This research was conducted from a feminist perspective that sought to center the voices of participants. Feminist research seeks to address power imbalances within research processes while generating knowledge to improve women's lives (Brooks & Hesse-Biber, 2006; Letherby, 2003). Feminist research challenges positivist notions that research is objective or that “facts” establish human behavior (Oakley, 1998, pp. 710–714) and is underpinned by key practices including reflexivity and reciprocity. Reflexivity requires that researchers reflect on the intersecting power hierarchies they occupy (Wickramasinghe, 2010). As an Australian-educated woman from a Sri Lankan background, with a history working in humanitarian settings and an affiliation with a British university, my power within the setting of Jordan constantly shifted and was renegotiated as I interacted with Syrians. Intersections between gender, race, and age mediated how I was perceived, at times permitting disclosures of difficult life experiences such as violence and racism, due to the perception that I was nonthreatening and “trustworthy.” In other cases my “outsider” status meant that I had to prove myself to participants. While I spoke conversational Arabic, I was often dependent on the presence of a research assistant, who spoke the language fluently and acted as an intermediary. In my research, engaging in the feminist practice of reciprocity involved disclosing personal information, sharing food and supporting refugees in obtaining information or services from humanitarian actors. Taking an “ethics of care” approach was part of my commitment to feminist research. This meant prioritizing the interests and concerns of my participants and not being solely fixated on gathering data (Malkki, 1995, p. 51).

Relationships were built with refugees through six participatory photography workshops held in three local humanitarian organizations over a 5/6-week period, with approximately 34 women and nine men attending regularly among others who attended sporadically. The local humanitarian organizations invited participants to attend the workshops, which were facilitated by the researcher and a translator. Workshops were an opportunity for socializing, eating together, and discussing daily experiences of life in Jordan using photography. The humanitarian organizations did not have a role in the sessions, ensuring participants felt comfortable to share their perspectives comfortably, including the challenges of living in Jordan. While there were passing references to marriage during photo elicitation exercises, these were limited to discussions about differences in how participants spent their time in Syria and Jordan. Participants did not discuss marriage or weddings in depth in workshops, however, detailed discussions of marriages and weddings occurred in interviews that followed the workshops. Workshop participants were invited for individual semistructured interviews or life story interviews, which occurred in refugees’ homes, cafes, restaurants, parks, or in the same organizations where workshops were held. In total, 20 semistructured interviews were held with 15 women and five men, as well as 10 life story interviews with seven women and three men. Life story interviews occurred over several sessions covering specific periods in participants’ lives: childhood, adolescence, adulthood, leaving Syria and life in Jordan. The discussion began with the question: “What did it mean to be a girl/boy growing up in Syria? Can you tell me about your childhood,” and used a similar question structure to invite narratives about later life stages. Additionally, 10 interviews were held with local and international humanitarian actors—though these interviews are not discussed in this particular article. Interviews and FGDs were audio recorded and transcribed into English, with support from research assistants. Transcripts were anonymized to ensure confidentiality.

This article does not seek to generalize about Syrian marriages; ethnographic accounts may not easily “translate” to generalizable data or lessons for humanitarian actors due to their focus on depth rather than breadth (Grabska, 2014, p. 193), nor are ethnographic accounts merely stories. Ethnographies are limited to the particular context and event they describe, “but at the same time reveal general features of human social life” (Hammersley, 1990, p. 598). In drawing attention to the complexities of studying marriage in a particular setting, this article provides a counter-narrative to dominant representations of Syrian marriages, situating these accounts within lived experience (Davis & Craven, 2016, p. 99; McIntosh & Wright, 2018, pp. 8–9).

## Findings

The article explores two key themes to demonstrate the diversity in experiences of marriage among Syrian women and men. The first theme explores the role of multiple influences in decisions about marriage, challenging the idea that older men (particularly fathers) are always the decision-makers about marriage and contributing insights into women's agency and decision-making. The second theme explores violence and love in marriage, and resistance to proposed love marriages, situating these accounts in both the predisplacement and displacement periods to challenge dominant narratives about Syrian marriages.

### Multiple Influences Over Marriage Decisions: Mothers, Young Men, and Women Themselves

Within analyses conducted by humanitarian actors and even academic literature, fathers are often presented as key decision-makers about marriage (Al Akash & Chalmiers, 2021). However, my research findings highlight a more complex picture of marriage decision-making, firstly through the role of women, which emerges in accounts of mothers in arranging and approving marriages. The accounts of Zubeida, who is now in her 60s, illustrate this influence. Zubeida, who is a family matriarch, lives with her husband, and their children and grand-children, sharing multiple rooms in an apartment block. During interviews, Zubeida alternated between discussing her life experiences, giving advice to her daughter-in-law (who was also present for some interviews), or sending her daughter-in-law on various errands. Before Zubeida was engaged, she had many potential suitors, but her future husband, who was in a secret relationship with her, tried to prevent other suitors from engaging with her family, by pretending to be promoting the interests of Zubeida's mother:

He ran after the suitors who asked for my hand and told them not to marry me and that my mother had sent him to tell the suitors her maternal cousin wants her. He didn't let them! When my suitors came, he would be on full watch mode …. ‘Her father wants her to get married but she [her mother] doesn't want you to come close to her!’

She further clarified the role of her mother in the marriage process: “If the mother doesn't accept this person then it is impossible for something to happen.” Her future husband recognized that there was societal acceptance of the mother arranging marriage, and sought to take advantage of this norm to deter his competitors. His actions reflected the fact that the mother was the one holding power. Zubeida attributed her mother's influence to her personality: “Even, till now, most of the mothers with an overly strong personality, if she doesn't agree to something then it's impossible for it to happen.”

The role of mothers in influencing marriage has also been documented in research within the region (Ghannam, 2013; Meneley, 2016), as well as in Syria. Kastrinou's (2016, pp. 100–113) study among the Druze discusses the role of the mother of the groom in the marriage negotiation process as well as wedding rituals. Salamandra (2006, pp. 155–156), in her research among higher-class Syrians in Damascus, discusses how women competitively display themselves to older women as part of the marriage selection process. Mothers identify potential girls for their sons to marry based on this process. One other research participant, who experienced this behavior as a single woman in Homs before the war, also reflected on the pressure to “display” herself to mothers in order to gain their approval:

Everyone told me, ‘Go to dance, you are young and have a nice outfit and you don’t dance?’ Some said, ‘No one will engage if you don’t dance’. It's an ignorant mentality. Some girls even danced but didn’t take the attention of the mothers …. The dancing is for the mothers. ‘She moves a lot, she seems good’.

Another woman shared a similar experience: “…when [a] mother comes and sees you in the street and admires you (laughing), like as if I am sitting under her mood …. I’m against this way.” Here, the idea is that older women exercise power in choosing younger women for their sons. Being “under her mood” signifies being subject to her preferences.

This is not to say that it is solely women who influence the marriage process. Importantly, what emerged from this research is that a young man may exercise varying levels of power over female relatives more broadly while “learning to become a patriarch” (Joseph, 1994a, p. 52). Fuad, who was aged 19 at the time of the interview, told this story about his aunt. Fuad's extended family are very close; his aunts (on his father's side) play a particularly important role in his life, emerging in many of his accounts. Fuad explained that when he was 15, he would supervise his 20-year-old aunt's interactions with her boyfriend. His aunt had loved someone from the third grade until university:

She had used a girl's name for him on her mobile. I found out about this and always used to take her phone and change the password and hide it. Grandma used to come and beg to open her cell phone. When she would go up to the roof to talk to him, I would follow her, and I didn’t let her talk to him.

In this account, Fuad deliberately inserted himself into the situation, to follow her when she was on the phone and to change her phone password, as a way of policing her behavior, despite being her young nephew. Fuad also took on this role with his sister: “My sister, if anyone wants to love her, or talk to her, she tells me. I know everything about her. That was our relationship since the days of Syria.” He added that he also has a female cousin who is close to him and tells him which men talk to her. A factor that may be relevant here is that his older brother is in love with this cousin, though nothing has officially been settled. This could be the reason that the cousin talks to Fuad about other men who are interested in her and allows him to engage these men on Facebook. Fuad has taken on a kind of intermediary role in policing who comes into contact with his cousin. Here it is unclear what the role of Fuad's father or other males in the family is, especially given Fuad's young age, and whether his actions toward females are viewed as competition or as an extension of the older patriarch's power (Joseph, 1994a, p. 62).

Other participants also referenced the idea of a brother protecting a sister in relation to marriage as a practice that was not the result of displacement. Roula's brother, who is 8 years older than her, threatened to shoot the man she loved. She went to her father but he agreed with her brother's decision; he was scared his son would get into trouble if he hurt her suitor. She was forced to let go of this relationship: “Can you accept your love to be shot?” This brother also hit Roula because of her love for this man which resulted in her father shouting at him, but Roula still had to give up this relationship.

However, the role of mothers and young men in influencing marriage does not deny the ability of girls and women to make their own decisions about marriage—or the role of women in making decisions about the marriages of young men (Zbeidy, 2020). Participant accounts about relationships with the opposite sex, marriage proposals and courtship processes challenge what might be termed typical representations of marriage as forced upon girls and women in the Middle East. Instead, interviews with Syrian women and men show how women challenge and negotiate around expectations that they should marry, making choices that allow them to follow their own aspirations first. Fuad, for example, first loved a girl when he was in Syria, as a teenager. He sent her love letters which she ripped up and threw away. When he finally spoke to her, having only admired her from a distance, she told him “I’m not going to love anyone now,” explaining that she wanted to finish secondary school first, then she would “start thinking of these things.” Amira, a young woman, told me her priority is her education. Aged 21 and unmarried, she is seen by her extended family as a “spinster.” They are shocked that she has refused prospective suitors, but Amira is resolute: “If everybody does it, I don’t have to follow them.” After many barriers to continuing education in Jordan, she was finally able to begin undergraduate studies. She sees marriage as something that stops women from being able to use their skills: “[T]hey leave their study when they reach university, or after they finish university they get married and they sit at home. I consider this to be wrong.” Amira's perspective is influenced by her family background and social class. Her parents were very supportive of her decision to study; her father himself is a professor and his emphasis on education has influenced her. Her family has faced less economic pressure than other families during displacement, which is perhaps what has enabled Amira to choose education.

Women may choose to remain single despite facing economic pressure. As a different kind of example, Khadija became a widow at age 29 in Syria. Despite financial challenges, she refused to remarry and remains single now in her early 60s. When approached by suitors after her husband died, her family told them, “It's her choice to make, it's not our business.” Her husband's family also expected her to marry again, but she told them, “No, after my husband, no! … [H]e spoiled me, no.” She had agreed with her husband not to marry again; she felt that a second marriage could result in her not seeing her children if the new husband forbade this. This is an example of how even when facing economic pressure or the need for male protection, a woman may not necessarily marry but may choose to remain single. Her relatives did not force marriage but allowed Khadija to make a choice, which may also be tied to her age and status as a widow. Sometimes marriage may restrict rather than increase options, in this case potentially limiting Khadija's ability to care for her children and breaking a promise to her husband. By exercising agency in deciding not to marry, the example of Khadija illustrates how marriage may not always be the solution to strengthening a woman's position but may at times undermine it. This contrasts with other literature that emphasizes the importance of marriage for social position (Salamandra, 2006; Taha, 2020).

For others, the bad experiences of married friends were a strong reason to think carefully about how marriage might threaten women's interests. Bana, a young woman, said that she has a friend whose husband does not treat her well. This husband also has a health problem preventing them from having children. She explained that this friend is “between two fires”: a disappointing marriage without children versus divorce and the “failure story” this will mean for the family. This example made her rethink her views on love: “[H]ow did he love her? He saw her when she was coming from school, and he was tailing after her until he engaged her. Love is not everything.” She added: “Some people, there are people in my age, that are married and their life is depressed. Their life, *khalaṣ* [that's it], that's it…. Live your life. Life is beautiful…. Things, there are things to make you happy, that will make you richer than having a hundred men.”

Bana's views on love and marriage echo the focus on pragmatism in marriage decisions (Abdallah, 2009), and were particularly striking for their honesty yet cynicism, which stem from her own life experiences. She faced significant challenges during adolescence, including racism and exclusion at school. Bana has darker skin than many Syrians and was bullied at school because of this, experienced name-calling and taunts from other children. She felt that these experiences helped to develop her strong personality and determination to work and study. In Jordan, she works as a teacher. She said that when she marries, she will set boundaries: “From the beginning, I [will] tell him, ‘I have my own privacy, I have my own friends. I can't forbid, abandon my friends, they have to come to visit me, I go out with them’.” She described a husband as “dough”: “It's like whatever you make from him, you make from him. If you raised him bad if you taught him correctly….” For her, this was about training the husband so that he knew his place from the outset: “[W]hat you let him used to, get used to, he will get used to.” This kind of strategic approach to marriage echoes the pragmatism of other literature documents about women's marriage decisions (Abdallah, 2009), however, extends beyond the choice of who to marry, to how a husband is to be managed. It is possible that this attitude to marriage is made possible by the fact that Bana has a job and the freedom to be more independent. Her experience growing up in a household where her brothers were working in the Gulf and the female family members managed the household affairs may also contribute to her sense of self-sufficiency and confidence. Bana's account challenges somewhat the notion of “waithood” (Singerman, 2006); instead, her experience has an active component like others have found in refugee settings (Brun, 2015) reflecting something more like “waiting willfully” (Eisenstein, 2021). Bana is active in the present, supporting herself and consolidating her views about marriage.

Another participant, Lubna, focused more on the concept of reciprocity: “If he spoils me, I’ll spoil him. If he doesn’t spoil me, I won’t spoil him.” She and Bana chatted about this in a joint interview:

Bana: If he gave me a gift, I’ll give him a gift.

Lubna: No I’m the opposite of her opinion. What if he didn’t give? I’ll give him a gift and after that …. If he didn’t bring a gift then he’ll never see any gift ever again….

Lubna's family recently experienced her sister's divorce and returning to the family home, which may explain her more calculated approach to receiving gifts. As the two young women spoke, they giggled about how they would engage with a future husband, including drawing boundaries to manage a husband's behavior.

The accounts above suggest decision-making about marriage is complex and builds on existing research that suggests that marriage is a “collective effort” (Zbeidy, 2020, p. 144). While the role of Syrian mothers in influencing marriage is a longer-term practice observed in previous research (Kastrinou, 2016; Salamandra, 2006; Zbeidy, 2020), scholars observe that women may engage in arranging marriages on behalf of male family members—complicating the idea that women have final or complete decision-making over marriages or may arrange marriages for young men (Zbeidy, 2020). In these accounts, the influence of young men emerges as a potential avenue to understand how patriarchal power operates within families, building on the work of Joseph (1994a) to position such decision-making as not necessarily being a result of displacement, but part of an established social practice. The accounts in this article contribute insights into how the influence of mothers and young men does not negate the decision-making of girls and women about marriage. Within what might be viewed as seemingly static gender norms, education status, family history, and pragmatic decision-making also shape women's choices. Contrary to dominant representations of Syrians, marriage may not be the sole aspiration of women or their families; marriage may not always be perceived as a positive and in some cases may undermine a woman's life choices. As such, despite facing economic and protection risks, women may still choose to remain single. As discussed in the next section, the complexities related to marriage decisions extend into marriage itself. Marriage may involve violence, marriages may also involve love, and proposed marriages based on love may be resisted by others.

### Violence in Marriage, Love Within Marriage, and Resistance to Marriage

In my research, the concepts of violence and love were embedded within narratives about marriage. While representations of Syrian marriage often involve descriptions of women's experiences of violence during displacement, the examples below focus on both the predisplacement and displacement period, rather than displacement alone. Alongside the more common narrative about men using violence against their wives and marriage characterized by tension, it is perhaps inevitable narratives of love are less visible. Within accounts of violence, love also emerges, complicating assumptions about power and the nature of marital relationships as solely functional. Plans for marriage based on love may sometimes also face resistance, such as pushback, negative reactions or even threats.

The concept of violence within Syrian marriage is evident in dominant humanitarian narratives about displacement, however, what is less clear is the dynamic between predisplacement and displacement violence. My life story interviews with one participant, Eman, demonstrate this trajectory of violence. Before displacement, Eman already had a difficult relationship with her husband, characterized by violence. Her husband is her cousin, who lived in the same village as her and was essentially raised by her mother. Eman did not complete her education—her father stopped her from going to school after the sixth grade. She described a mismatch between what she imagined marriage would be or what she saw on TV, and what it actually is: “[R]eality is different … responsibility to the husband, responsibility to the in-laws. This you should say and this you shouldn't say, this you have to do and this you don't do…” Eman described how her life changed after marriage: “There is no freedom anymore. *Yaʿnī* [this means that], a girl's life is different from a woman's life.” In Jordan, her husband's health has worsened, affecting his ability to work and increasing the economic pressure they face. Eman blames his illness for the violence she has experienced; she feels his illness causes him to be violent. In both Jordan and Syria, her family became involved in the conflicts with her husband, interfering perhaps because he is also their relative. During displacement, perhaps in response to the greater opportunities for socialization Eman experiences compared to their small village in Syria, Eman's husband monitors her interactions with others. He accompanies her on visits to friends. Eman lost her smartphone, but he would not allow her to buy a new one. Not having WhatsApp or access to the internet restricts her ability to interact with friends and family.

Violence may also emerge in the form of control, including coercion and manipulation, which may occur through the threat of “verbal divorce,” or “triple *talāq*” that is sometimes practiced as part of Islam. Verbal divorce involves saying “*talāq”* to your wife three times. Although not seen as valid in some countries, it is agreed by some legal scholars to be a legitimate way of divorcing someone (Ahmad, 2009). Dina, a young woman, also faced a difficult marriage including experiences of physical and emotional violence. In Syria, her husband regularly threatened divorce: “He may have thrown 60 divorces on me!” These “oaths” were usually a result of Dina's sisters-in-law (his sisters) making complaints about her. Dina went too far as to ask a Sheikh in Syria about these divorces; he said verbal divorces did not count, because they were not documented. Interestingly, in Jordan, her husband did not verbally divorce her. Dina attributes this to the fact that they lived together with his family in Syria, but in Jordan, his family live far away and exerts less influence on their marriage. Despite the gender order which positioned Dina as subordinate to her husband, the patriarchal pull of family was lessened during displacement due to distance. The change in context translated to her husband's family having less influence, improving marital relations.

In other cases, violence may emerge later, exacerbated by an interplay of factors including neoliberal economics and family dynamics, and sustained by the belief that fate or destiny shapes people's lives. Aya, another young woman, also experiences violence from her husband. Before coming to Jordan, Aya was promised in marriage to someone else, however, fell in love with a Syrian man in Jordan and ended up marrying him in Jordan. In her research among Palestinian refugees, Stéphanie Abdallah (2009, p. 58) suggests that exile changes people's expectations: “Their ideas about life and marriage are more pragmatic. Marriage for love is seen as utopian and not expected.” In this example, it perhaps would have been “pragmatic” to abandon the new relationship in favor of the person she had already been promised to, but Aya pursued love instead. She had only been in Jordan for a short time, but she chose the person she loved instead of the less complicated option. Aya laughed as she observed, “After love then the bear comes out!” This comment about the bear is an idiom—love (*ḥubb*) and bear (*dubb*) have a similar sound. Over multiple interviews, Aya described how her life completely changed after marriage: what began as love turned into a relationship characterized by violence. She and her husband face significant financial pressure because it is difficult for her husband to find work. Aya is not allowed to go out unless her husband gives his permission, even to visit her mother, who lives a short walk away, though sometimes she sneaks out. She said: “[H]e likes to control me, he doesn't let me go out … He doesn't want me to go to my family, not to breathe air.” Her husband also finds other ways of regulating her behavior, including confiscating her phone and threatening to remove WhatsApp, affecting her social interactions. Aya has also experienced violence from her husband on multiple occasions, including in front of his parents, which was particularly humiliating. Aya however seemed to justify this behavior, reiterating the idea echoed by many women, that men are under pressure in Jordan and should not be bothered: “If someone can't let it out on his wife, who is there for him to let it out on?” Through tears, as she recounted the many challenges she faces, including multiple threats of divorce, Aya laughed, saying, “But it's *naṣīb* [fate/destiny]. Every human being takes their *naṣīb* in this world. *Hamdu li’llāh* [Thanks be to God] for everything.” *Naṣīb* is a cultural and religious concept referring to fate or destiny, which is recognized as playing an important role in shaping decision-making (Alkhudary et al., 2021; Hassan et al., 2015). For Aya, *naṣīb* keeps her in her marriage. It is difficult to know whether *naṣīb* is what is actually stopping Aya from leaving her husband, or whether other factors matter, like the shame of divorcing or the impact on her child.

In some cases, it is not violence that emerges within accounts of love, but the notion of resistance as an external threat to love. Within existing literature about Syrian marriages, there is sometimes reference to how individuals might resist plans for their own marriages, but few accounts of how other family members might resist love marriages (Abdallah, 2009; Kandiyoti, 1988). This article thus contributes to new knowledge about the varied reasons for resistance to proposed love marriages. One participant talked about the four male suitors she had before marriage. When she finally married, one suitor wanted to shoot the person she eventually married—in this case, new love was threatened by a previous relationship of love. Resistance also emerges in Fuad's experience. Fuad met a Syrian girl in Jordan a few years ago. His family do not support them getting engaged because she has not finished her studies and is in the 10th grade. Her family, although they are quite poor, want someone much richer than Fuad for their daughter. Fuad's mother is on his side, but his father has resisted their relationship. One tense argument with his father, mother and aunt resulted in his father slapping him, and Fuad running away to another aunt's house. Fuad was angry: “I swore on the Quran that I will marry against their wish.” He added: “I only feel I want to challenge them. Some days I thought of going back to Syria. Because I’m so tired of the subject […] Then they will let me not just marry her, but let me marry two!” His strategy for getting his way was about waiting—wearing his family down and letting enough time pass for the girl he loves to finish school. In this account, not just parents but the immediate family were involved, demonstrating the broader role of Fuad's aunts in his life. Resistance operated from two levels: his family were concerned about the girl's age, and her family because of his lower economic status.

Resistance to marriage can be caused by other reasons too. Ibrahim and Yasmeen initially faced resistance to their marriage because theirs was the first marriage “within the family” (between cousins) and other relatives wanted Ibrahim as a spouse for their daughters. This engagement resulted in both of them receiving threats:

Ibrahim: Even the day of my engagement, I was threatened to be killed! … They threatened me on phone more than once, and called her family to threaten her, too. But unfortunately, I knew who was it. They were from my family. Because I didn't take from them …. They wanted me to take their daughter. Even another woman told my wife, ‘You took the person who I love!’

Yasmeen: He's the one who took me, I didn't take him!

Yasmeen was the youngest of all the cousins; her other cousins were, at least in the eyes of their parents, more ready to be married. Ibrahim was given a gun by one uncle and told to shoot anyone who came to hurt him. Ibrahim was able to defuse the situation once he knew who was making the threats. In this example, threats of violence stemmed not from the shame of choosing the wrong spouse, rather related to competition between women, which Salamandra's (2006) research has already found among Syrians. Cousin marriages had not occurred in the family yet. Yasmeen joked, “He was contagious, after that the whole family!”

Resistance from others may also take more subtle forms. Khadija, who is now in her 60s, was married at the age of 12. This marriage was however a happy one: “Every day with him I was a new bride. He loved me so much.” Her husband, who was aged 17, would buy her sweets and other delicious foods. He made her a swing and would push her on it. Although the marriage was arranged by the *mukhtar* of the tribe, who was also her grandfather, it became a relationship of love which Khadija described as “honey” before the “onions” that followed when her husband unexpectedly passed away. In this example, love was present within an arranged marriage—building on literature that counters the false dichotomy between arranged and love marriages (Zbeidy, 2020). The love between Khadija and her husband was however perceived as a problem by their broader family. For example, Khadija's husband was flexible in allowing her to visit her family, but his sisters became “crazy” when they heard about this:

They say like, ‘He is like a ring in your finger. What did you do to him? What did you do to him?’ So, they didn’t know that we are just getting along together.

This Arabic idiom “He is like a ring on your finger” refers to the wife controlling him: a variation of the saying in English, “She has him wrapped around her finger.” As Khadija recounted this story, she laughed, “They thought I did something to him so he would love me!” Khadija's sisters-in-law reacted to Khadija being given freedom by her husband and assumed this was linked to her exercising power over him, rather than the love between them. Here, the sisters-in-law recognized that it was possible for women to exercise power over their husbands. In this case, the perception that this was occurring was enough to cause problems, resulting in Khadija experiencing ongoing conflict with her sisters-in-law.

However, resistance is not always something to be managed like in the case of Yasmeen and Ibrahim, or something that impacts relationships in the long-term like in Khadija's experience. At times resistance may be mobilized and leveraged to gain power, like in Zubeida's experience. She met her future husband as a teenager. Her father felt she needed to be older before engagement and he also preferred her to marry a family member rather than a “stranger.” After a few years, however, her father relented. During this time, Zubeida used the resistance to the marriage to get what she wanted from her future husband, taking advantage of his love for her: “all he wanted to do was please me.” She saw this as an “opportunity” to get her way, even during their engagement, resulting in her fiancé agreeing that they could live separately instead of with her in-laws (as was the custom). She chose furniture for their apartment herself and secretly arranged meetings with her fiancé, deviating from gender norms about how future brides should behave.

In this example, Zubeida capitalized on the love of her future husband, using it to exercise agency. Later, as a refugee in Jordan, she continues to use her influence to achieve her wishes, directing the actions not just of her daughters and her daughter-in-law but also of the male family members including her husband, using her age to challenge seemingly fixed gender norms and social structures (Rabo, 2008; Rugh, 1997). This aligns with Joseph's (1994b, p. 282) analysis of how family dynamics and age may place women in “privileged positions.” The love between her and her husband first laid the foundation for her to begin and then continue to exercise agency. Being the oldest female (Kandiyoti, 1988) provided Zubeida with the opportunity to continue exercising power.

Khadija and Zubeida's experiences accentuate how love may be present within marriage over the long term. Another woman, Hadiya, met her husband on a bus; he held her luggage. He found out that she regularly took this bus by making inquiries at the bus office and manoeuvred his way to sit next to her each time. She explained that in her whole family, and even in the village she lived in, people married for love. Hadiya felt that in the countryside it was easier to marry for love, but that people in the city did not have the approval of their families and could not do so. Her perception may also be linked to her socio-economic position; her family were relatively well-off and highly educated, perhaps allowing greater freedom to choose love. Hadiya's accounts also challenge common representations of expectations of women within marriage. She ran her own medical practice and worked in a government hospital prior to the war, and for her, the most difficult part of living in Jordan has been dealing with her children. In Syria, her parents and in-laws helped with childcare, but in Jordan, without work, she spent all day with her children. The tone with which she described her frustration was similar to how refugee men talk about not being able to work: “I am … embarrassed because I am not working and all my siblings are sending [money] for them [her parents]. They give them and I am…” Her voice trailed off. For Hadiya, being unable to contribute financially to her parents (who she and her family live with in Jordan) was uncomfortable. However, for others, the notion of women working is less acceptable. One older man, of a lower socioeconomic status than Hadiya, commented, “A man who sends his wife to work while he sits at home is a *nāmūs*!.” The word *nāmūs* has two meanings, referring to a gnat or mosquito, or, more colloquially referencing the right of a man to ensure virtue or honor of his household. This dual meaning is invoked to refer both to the failure of nonworking men to preserve the honor of their families, and to the fact that this behavior makes them like insects. He explained that such a person is “not a real man, he has no chivalry” and is “not man enough.” In these examples, varied expectations for how women and men should spend their time within a marriage challenge depictions of married Syrian women as passive and lacking agency (Gallagher, 2012; Rabo, 2008; Zbeidy, 2020) and reinforce the idea that gender norms are not static but are context-dependent (Inhorn, 1996).

While it may be more socially acceptable for women rather than men to discuss love (Khuri, 2007, p. 25), in one interview, both a wife and husband admitted to love. During a joint interview with Jamal and his wife, Yara, the atmosphere changed when I started to ask questions about their marriage. Jamal and Yara started smiling and laughing, along with their daughter. Almost every sentence was followed with laughter and giggling. Yara said, “It was love, sister!” when I asked how their marriage came about. She laughed saying, “I still love him and die for him” and then wanted me to ask if he still loved her, but he voluntarily said, with a big smile on his face, “I love her, yes.” She described their elaborate wedding: “He gave me a piece of chocolate to eat during the wedding … He put it in my mouth” and everyone erupted in laughter. She added, “This is normal, there's nothing wrong with it.”

These accounts of violence, love, and resistance to love marriages challenge dominant current representations of Syrian marriages. Before and during displacement, powerful ideas keep women in difficult marriages, specifically the idea that a husband is behaving this way due to an “external” stressor (influential family members or illness), or the idea of *naṣīb*, which makes people feel like their life scripts cannot be changed. These difficult relationships between husbands and wives also demonstrate what Aitemad Muhanna (2013, p. 17) found in her research in Gaza, that love and control “may be enacted in parallel.” In these accounts of marriage, love emerges as a rationale for marriage as well as a threat (Abdallah, 2009, pp. 58–59). Resistance to love may take various forms, as couples defy expectations by family members, are deemed too young, or face strained family relationships. In some cases, resistance may provide opportunities for those usually lacking the power to exercise power, however, women also find other opportunities to exercise agency in ways that challenge common assumptions about patriarchy and Islam in this region as well as men's expectations of them (Gallagher, 2012; Mahmood, 2001; Rabo, 2008; Rugh, 1997; Zuntz et al., 2021), for example, through seeking to work. Along similar lines, men may challenge notions that love is not to be spoken about by expressing feelings for a wife (Khuri, 2007). The notion that marriages in the Middle East context occur for pragmatic or functional reasons can be challenged through accounts of loving marriages.

## Conclusion

The article makes important contributions toward understanding the lived experiences of Syrian women and men, challenging dominant representations of Syrian marriages. The depiction of the social practices and norms related to marriage in this article provide important social and historical context which has implications for policy actors, including humanitarian actors (McIntosh & Wright, 2018). The findings demonstrate how perspectives on marriage differ based on individual experiences, gender, age, social class, economic status, upbringing, and education. These nuances enable different narratives to emerge, for example, girls choosing to delay marriage to pursue education or perceiving a husband as someone to be strategically managed in order for a wife to benefit. The fact that women may exercise agency to choose not to marry because marriage may hold the potential to undermine them, represents an important contribution of the study. This finding contrasts with existing literature in the region which links marriage to improved social status for women (Salamandra, 2006; Taha, 2020), instead suggesting that there are situations where women may decide marriage is not the best option. In these accounts, women's role in choosing (or not choosing) spouses also places them in positions of power, which is a less-visible narrative about Middle Eastern women both in academic literature as well as in humanitarian reports. The findings in this article also demonstrate the role of young men in policing relationships of female relatives, which represents a topic requiring further research. In these accounts, attention is also drawn to women's own decision-making and maneuvering around norms for marriage.

This article also reinforces existing research on violence and control within Syrian marriages. The idea that violence is due to external forces is similar to humanitarian narratives which blame displacement for violence (Buecher & Aniyamuzaala, 2016, p. 14; CARE Jordan, 2013, p. 31; UNFPA, 2019, p. 27), negating the role of patriarchy or neoliberal forces in shaping violence. Blaming fate (*naṣīb)* may result in women feeling they cannot exercise agency to leave a violent relationship. Accounts of love also emerge from the findings on marriage, reinforcing the idea of love being viewed as a threat (Abdallah, 2009, pp. 58–59), challenging the notion that love does not exist in arranged (or early) marriages, and suggesting love may be used to a woman's advantage to help her get what she wants. This article contributes to the limited literature on resistance to love marriages (Abdallah, 2009; Kandiyoti, 1988) by exploring ethnographic accounts of resistance being a barrier to marriage, as well as how resistance may be leveraged to improve a woman's position. Accounts of marriage based on love contrast with singular humanitarian narratives about marriage occurring for economic or protection reasons, challenging the notion that love is a “Western” concept.

While these findings emphasize women's agency and influence, the fact that women may exercise power does not negate patriarchy (Inhorn, 1996, p. 8) nor its complex interactions with other forces (Abu-Lughod, 2013; Kandiyoti, 1988; Patil, 2013). Instead, “gender relations are less given than made, unmade, and remade” (Inhorn, 1996, p. 19).
